# Effect of Emailed Messages on Return Use of a Nutrition Education Website and Subsequent Changes in Dietary Behavior

**DOI:** 10.2196/jmir.9.3.e27

**Published:** 2007-09-30

**Authors:** W Gill Woodall, David B Buller, Laura Saba, Donald Zimmerman, Emily Waters, Joan M Hines, Gary R Cutter, Randall Starling

**Affiliations:** ^6^University of AlabamaBirminghamALUSA; ^5^The Children’s HospitalDenverCOUSA; ^4^Colorado State UniversityFort CollinsCOUSA; ^3^University of Colorado at Denver and Health Sciences CenterDenverCOUSA; ^2^Klein BuendelIncGoldenCOUSA; ^1^University of New MexicoAlbuquerqueNMUSA

**Keywords:** Internet, diet, adult, behavior change, nonresponse, electronic mail, attrition, usage

## Abstract

**Background:**

At-risk populations can be reached with Web-based disease prevention and behavior change programs. However, such eHealth applications on the Internet need to generate return usage to be effective. Limited evidence is available on how continued usage can be encouraged.

**Objective:**

This analysis tested whether routine email notification about a nutrition education website promoted more use of the website.

**Methods:**

Adults from six rural counties in Colorado and New Mexico, United States (n = 755) participating in a randomized trial and assigned to the intervention group (n = 380) received, over a period of 4 months, email messages alerting them to updates on the website, along with hyperlinks to new content. Update alerts were sent approximately every 5 weeks (each participant received up to 4 messages). Log-ons to the website were the primary outcome for this analysis.

**Results:**

A total of 23.5% (86/366) of the participants responded to at least one email, and 51.2% (44/86) of these participants responded to half of the email messages by logging on to the website. Significantly more log-ons occurred on email notification days compared to all other days (OR = 3.71, 95% CI = 2.72-5.06). More log-ons also occurred just after the notification but declined each day thereafter (OR = 0.97, 95% CI = 0.96-0.98 one day further from mass email). Non-Hispanics (OR = 0.46, 95% CI = 0.26-0.84), older participants (OR = 1.04, 95% CI = 1.04-1.06), and those using the Internet most recently (OR = 0.62, 95% CI = 0.51-0.77) were more likely to log on. Responders to the messages had a more positive change in fruit and vegetable intake (mean change = +1.69) than nonresponders (+0.05), as measured with a food frequency assessment (adjusted Spearman partial correlation coefficient = 0.14, *P* = .049). Compared to nonresponders, responders were more likely to be non-Hispanic (*P* = .01), older (*P* < .001), and had used the Internet more recently (*P* < .001).

**Conclusions:**

Messages sent by email appeared to promote a modest short-lived increase in use of a disease prevention website by some adults. Those who responded to the messages by logging on to the website may have been influenced to improve their diet.

## Introduction

Evidence is mounting that Internet technology can be used to reach at-risk populations with Web-based disease prevention and behavior change programs. However, attrition and low use of these websites appears to be a common occurrence when they are used in community settings where participants are not “required” to use them. Surveys indicate that people are using the Internet to obtain health information; however, some immediate acute need, most commonly a real or potential health problem, seems to be the primary driver rather than the need for preventive information [1]. Unfortunately, strategies to improve exposure to health communication have not been investigated as often as strategies to influence health behavior [2].

It is a well-established principle in media effects research that audience activity determines the impact of media messages [3-5]. Exposure to media messages is fundamental to achieving effects, whether it be for news coverage, entertainment content, or a health communication program [2,4,6-8]. Audience members are selective in their choice of media and the content within media [9], and selective exposure has been demonstrated in new media such as the Internet [10].

In the case of the Internet, the choice of websites is essentially endless. The study website must compete with a large number of health-related websites for participants’ attention, not to mention the more than 350 million listings on Google related to nutrition, fruits and vegetables, cancer prevention, and diet—the topics of the current project—as of August 2007. In this situation, it may be that effective methods of increasing website use involve reminding adults about the website, providing hyperlinks that make it easy to visit with a single mouse click, and showing users what new content has been recently published to the website.

Beyond the conceptual issues surrounding why and how often individuals use the Internet, attrition and low website use threaten the internal validity of medical Internet research [11]. The experimental comparison in a randomized trial can be compromised when a substantial number of study participants do not visit the website being tested. Researchers often must rely on association between website usage, which is self-selected, and disease prevention outcomes rather than the randomly assigned comparison between treatment groups. In these cases, the possibility that a third variable(s) determines both greater website use and changes in prevention behavior undermines conclusions that websites are effective. It is also possible that website use is so infrequent in a trial that no association can be detected between treatment group or website use and health behaviors. In both scenarios, a potentially effective website is considered a failure without giving it a chance to demonstrate its full benefits.

In a recent community-based trial evaluating a nutrition education program, periodic messages were sent by email to notify participants of new content posted to the website and to remind them to visit the site. This paper reports the analyses of the effect of these email notifications on promoting visits to the website.

## Methods

### Context

The data reported in this paper were collected as part of a randomized trial to evaluate the effects of a nutrition education website promoting the consumption of fruits and vegetables. The main trial procedures have been reported elsewhere [12]. Relevant portions of these procedures are summarized here, and the content and methods for delivering the email messages to study participants and assessing usage of the website are described in full.

### Population and Sample

A sample of 755 adults in a six-county rural region spanning the border between Colorado and New Mexico in the United States participated in the main trial. Participants had to live at least 6 months or more in the region at pretest, be 18 years of age or older, and consent to participate. Data from the 380 participants randomized to the intervention group were analyzed for this paper.

Participants were recruited by 12 community outreach trainers from June 2002 to January 2004. Community outreach trainers were local adults recruited and trained to locate potential participants, introduce the study, obtain informed consent, conduct the pretest interview (using computer-assisted interviewing software), and assign participants to study condition (using a randomization computer program written by the project biostatistician). Community outreach trainers were blind to condition during recruitment, enrollment, and pretesting. When necessary, they also could provide basic computer and Internet skills training to participants. Community outreach trainers provided each participant with a unique user identification code (ID) to access the website, and this ID was used to track website usage. Project staff observed community outreach trainers during the trial and confirmed that they performed project tasks as trained [12].

### Trial Procedures

The main trial involved a randomized pretest-posttest controlled design, with individuals randomized to receive immediate access to the study website (intervention group) or to receive delayed access to it after the posttest (control group). At 4 months post-randomization, participants completed the posttest by telephone with professional telephone interviewers blind to the study condition.

### Nutrition Education Website: “5 a Day, the Rio Grande Way”

The “5 a Day, the Rio Grande Way” website contained content on the health benefits of fruits and vegetables; instructions for buying, storing, and preparing fruits and vegetables; and ways to increase fruits and vegetables in the family diet, particularly with children. The following were also provided on the website: information on gardening, recipes, and fruits and vegetables in season; a community directory that included organizations that sold fruits and vegetables or supplies for gardening in the six-county region; and a listing of health resources on the Internet related to fruit and vegetable consumption. The selection and organization of content areas on the website were based on social cognitive theory [13] and diffusion of innovations model [14] and were guided by expert advice and results from focus groups on nutrition and health information [15], evaluation of alternative message formats [16], and usability testing on the initial website structure with local residents [17].

### Content of the Reminder Messages

A total of 12 different messages were created and sent to participants announcing new content on the website. In each message, universal record locators (URLs) were provided, linking to the areas in the website containing the new content. These email notifications were designed to alert participants that new, important, and relevant information had been added to the site. Participants were invited to take a look at this new content, which, in some cases, was seasonal in nature. The messages were personally addressed to each participant, and the participant’s username and password were provided in the messages. The participants were reminded to contact the community outreach trainer who recruited them if they had any questions about the website or problems accessing it.

### Procedures for Delivering Reminder Messages

Approximately every 5 weeks, participants were sent a reminder message by email. The messages were sent to the email addresses provided by participants at trial enrollment. If emails were returned as undeliverable, the community outreach trainers attempted to contact participants and obtain an updated email address. The messages were sent from the central offices of the study. Corresponding to the number of updates on the “5 a Day, the Rio Grande Way” website during the 4-month observation period, most participants received 2 (35.7%, 136/380), 3 (32.1%, 122/380), or 4 (27.1%, 103/380) messages highlighting the updates. There were five participants who received only one message. (There were 14 participants not eligible to receive any messages because they were part of a pilot test of recruitment strategies.)

### Measures

Website use was recorded with a custom-made program running on the Web server. Each usage session was identified and linked to a unique participant using their ID. Number of log-ons to the website, total time spent on the website, and visits to various website features were recorded and totaled for the 4-month period. Also, the date each reminder message was emailed was recorded, and the number of reminder messages each participant was eligible to receive within the 4-month (120-day) period was determined. In the present analysis, log-ons to the website were analyzed because they indicated that a participant had visited the website. Also, log-ons displayed a high correlation with time spent using the website [12].

Change in dietary behavior was measured with a validated food frequency measure of fruit and vegetable intake conducted at pretest and posttest [18]; an item was added to assess consumption of common regional foods such as red chile, green chile, and salsa. Responses were converted to servings per day following Thompson et al [18]. Additional pretest and posttest measures assessed attitudes toward cancer and its prevention, support from others to eat fruits and vegetables, involvement in purchasing and preparing foods, experience using computers and the Internet, perceived self-efficacy for using the Internet, and social and demographic characteristics as potential predictors of response to the email remainders. Participants reported on their frequency of exhibiting 13 eating and meal preparation practices. Two composites with adequate reliability were identified using a principal components analysis with a varimax rotation: eating habits (pretest Cronbach alpha = .75, posttest Cronbach alpha = .69) and access habits (pretest Cronbach alpha = .66, posttest Cronbach alpha = .58).

### Statistical Analysis

Three analyses were performed in this paper to explore different aspects of the effect of mass emails on use of the experimental website and the response to it: (1) effect of email messages on log-on rates, (2) characteristics of participants who responded to the email messages by logging on, and (3) association of response to the email messages (by logging on) with dietary change. These analyses are explained in greater detail below.

### Effect of Email Messages on Log-Ons to the Website

To explore whether users were prompted to log on to the website after a mass email was sent, a generalized linear mixed model for a binomial outcome (a form of generalized estimating equations) with a logit link function [19] using PROC GENMOD in SAS was utilized (SAS version 9.1, SAS Institute Inc, Cary, NC, USA). In this model, subjects had an observation for each day between their pretest and posttest. The outcome of interest is a 0/1 variable, where 1 indicates that the subject logged on to the website that day. The independent variable of interest was a 0/1 indicator for whether or not a mass email was sent that day. The generalized linear model allows for a first-order autoregressive covariance structure to account for within-subject correlations where time (in days) is measured from the date of the participant’s pretest.

### Characteristics of Participants Who Responded to Email Messages

In addition to determining the effect that email alerts had on log-ons to the website, it was important to explore what participant characteristics were associated with response. Subjects were classified as responders if at any point during their study period (4 months) they visited the “5 a Day, the Rio Grande Way” website within 5 days of a mass email being sent. Associations between variables with this classification of response were examined using a logistic regression; odds ratios were derived to provide a measure of effect size. A multivariate model for predicting response status was created using a forward stepwise selection procedure. Results from this multivariate model are reported as factor effects using a Wald chi-square to test for significance.

### Association of Response to Email Messages With Dietary Change

The final analysis explored the relationship of the main outcome of the original study — change in fruit and vegetable consumption — to the response to mass emails. Using the same classification of responder as in the previous section, change in fruit and vegetable intake from pretest to posttest was correlated with responder status. In a separate analysis, the proportion of mass emails that the participant responded to was correlated with change in fruit and vegetable intake. All correlations utilized a nonparametric Spearman partial correlation, adjusting for variables in the final multivariate model predicting response status. A nonparametric test was chosen due to the non-normal distribution (excessive skewing) of the change in fruit and vegetable consumption.

## Results

### Description of Sample

Table 1 presents the demographic characteristics of participants randomized to the intervention group. They were predominantly female and married, ranged in age from 18 to 86 years, lived in multi-person households with children, and were long-term residents of the study region at enrollment. Nearly two thirds self-identified as Hispanic, and almost one tenth were Native Americans.

Hispanics had used the Internet less frequently (0 times = 20.9%, 50/239; 1-4 times = 10.5%, 50/239; 5-10 times = 5.0%, 12/239; > 10 times = 63.6%,152/239) than non-Hispanics (0 times = 10.8%, 13/120; 1-4 times = 5.8%, 7/120; 5-10 times = 2.5%, 3/120; > 10 times = 80.8%, 97/120; n = 379, *χ* ^2^_3_ =11.19, *P* = .01). However, among current users of the Internet, there was no significant difference by Hispanic origin in time spent on the Internet in a typical day (Hispanic mean = 7.0 hours, SD = 10.6; non-Hispanic mean = 7.2 hours, SD = 9.1; *t*_1,245_ = 0.17, *P* = .87), but fewer Hispanics had an email account (73.7%, 112/152 current Hispanic users; 89%, 88/99 non-Hispanic users; *χ* ^2^_1_ = 8.56, n = 251, *P* = .003).

About one third of participants had a high school degree or less education, and another one third had a 2-year or 4-year college degree or postgraduate education. Most of the participants had prior experience using the Internet, but nearly one third had used it 10 times or less (Table 1). Two thirds reported currently using the Internet at enrollment.

**Table 1:** Demographic characteristics and Internet experience of participants in the intervention group of the randomized trial (n = 380)

Demographics			No.		%
**Gender**					
	Male	47		12.4	
	Female	333		87.6	
**Age**					
	20 to 29	133		35.0	
	30 to 49	131		34.5	
	50 or older	104		27.3	
	Refused/missing	12		3.2	
**Hispanic origin**					
	Of Hispanic origin	246		64.7	
	Not of Hispanic origin	130		34.2	
	Refused/missing	4		1.1	
**Race**					
	American Indian / Alaska Native	36		9.5	
	Asian	3		0.8	
	Black	2		0.5	
	Native Hawaiian / Pacific Islander	1		0.3	
	White	133		35.0	
	None of these	173		45.5	
	Refused/missing	32		8.4	
**Education**					
	Eleventh grade or less	49		12.9	
	High school graduate / GED	82		21.6	
	Trade school or some college education	133		35.0	
	2-year or 4-year college degree	78		20.5	
	Postgraduate	38		10.0	
**Current marital status**					
	Married or living with someone	209		55.0	
	Widowed	22		5.8	
	Separated or divorced	54		14.2	
	Never been married	91		23.9	
	Refused/missing	4		1.1	
**Number of people in household (including subject)**					
	1	46		12.1	
	2	105		27.6	
	3	75		19.7	
	4	90		23.7	
	5 or more	64		16.9	
	Refused/missing	0		0.0	
**Number of minors in household**					
	0	157		41.3	
	1	77		20.3	
	2	96		25.3	
	3 or more	48		12.6	
	Refused/missing	2		0.5	
**Number of years subject resided in Upper Rio Grande Valley**					
	Less than 1	5		1.3	
	1 to 10	63		16.6	
	11 to 20	70		18.4	
	More than 20	232		61.1	
	Refused/missing	10		2.6	
**Number of times ever used Internet**					
	None	67		17.6	
	1 to 10	51		13.4	
	> 10	259		68.2	
	Don't know	3		0.8	
**Currently use Internet**					
	Yes	260		68.4	
	No	50		13.2	
	Never used	67		17.6	
	Refused/missing	3		0.8	

### Effect of Email Messages on Log-Ons to the Website

Overall, 23.5% (86/366) of participants responded to at least one of the email messages by logging on to the website within 5 days of when the message was sent. Only 6 participants responded to all of the messages sent to them. Of those who responded to at least one email, 51.2% (44/86) responded to half or more of the messages, while 48.8% (42/86) responded to fewer than half.

Participants were more likely to log on to the website on days when the messages were sent than on days when no email was sent (OR = 3.71, 95% CI = 2.72-5.06). This relationship remained evident when the analysis was expanded to include both the day the mass email was sent and the day after (OR = 4.11, 95% CI = 3.26-5.19).

**Figure 1:**
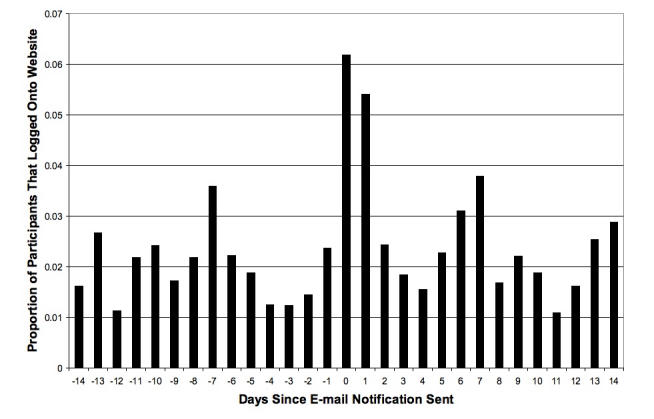
Average number of log-ons on 14 days prior to and after sending email messages to participants

However, Figure 1 shows that the effect of the messages was short-lived. The number of log-ons to the website appeared to return to the frequency seen before sending the messages by 2 days after the messages were emailed. In fact, the odds ratio associated with a 1-day increase in time since last mass email is significantly less than 1 (OR = 0.97, 95% CI = 0.96-0.98), confirming this declining rate (OR = 0.85, 95% CI = 0.82-0.88, for the decline over 5 days after email message was sent).

### Characteristics of Participants Who Responded to Email Messages

Several baseline characteristics (household size, number of children, education, race, Hispanic ethnicity, age, length of residence, gender), Internet experience (computer and Internet use, length and recency of Internet use, Internet self-efficacy, health information seeking self-efficacy, prior Internet training), and dietary patterns (readiness to change fruit and vegetable intake, diet responsibility, benefits of fruits and vegetables, dietary habits related to fruits and vegetables, comparison to peer intake of fruits and vegetables, family and friend support for eating fruits and vegetables) were tested for their association with visiting the website within 5 days of the email messages. The multivariate model predicting response contained three variables: (1) Hispanic ethnicity, (2) time since last use of the Internet (1 = within the past month, 2 = 1 to 5 months ago, 3 = 6 to 12 months ago, 4 = more than 12 months ago, 5 = never used Internet / not currently using Internet), and (3) age (Table 2). Non-Hispanic participants, older participants, and participants who has used the Internet more recently were more likely to log on to the website on the day of and the 4 days after the email messages were sent.

**Table 2:** Pretest characteristics significantly associated with responding to the email messages in final multivariate model

Characteristic	Odds Ratio^*^	95% CI	*χ*^2^	*P* value
Hispanic ethnicity	0.46	0.26-0.84	6.49	.011
Amount of prior Internet use^†^	0.62	0.51-0.77	19.68	< .001
Older age	1.04	1.02-1.06	17.51	< .001

^*^Odds ratio indicates amount of change in response to email messages associated with being Hispanic (as opposed to non-Hispanic); one-unit increase on Internet use scale, and 1-year increase in age.

^†^Scale is 1 (within the past month), 2 (1 to 5 months ago), 3 (6 to 12 months ago), 4 (more than 12 months ago), and 5 (never used Internet / not currently using Internet); odds ratio indicates amount of change in response to email reminders associated with one-unit change on this scale of Internet use.

### Association of Response to Email Messages With Dietary Change

Responding to the email messages was associated with dietary improvements. Participants who visited the website within 5 days after the message was sent reported larger increases in fruit and vegetable intake than those who did not (Spearman partial correlation coefficient = 0.14, *P* = .049; adjusted for ethnicity, age, and time since last use of the Internet). However, the partial correlation between the proportion of messages to which participants responded and increased daily intake of fruit and vegetables was nonsignificant (*ρ* = 0.11, *P* = .12).

## Discussion

Sending messages that highlighted new content published to a nutrition education website appeared to prompt about one third of adults in a community setting assigned to use the website to log on and visit it, as it did in hospital contexts [20,21]. However, the effect of each message was short-lived, with number of log-ons returning to pre-message levels within 3 to 5 days. Moreover, many participants responded to less than half of the messages. Thus, this strategy is, at best, modestly successful at increasing exposure to a disease prevention website.

### Selective Exposure to Disease Prevention Websites

Selective exposure to media arises because people have limited capacity to process messages. Attention is driven both by volitional processes under individuals’ control and automatic cognitive orienting systems [22]. Theories such as the Cognitive Mediation Model hold that volitional selective exposure is driven by needs and motivations [6,8] (eg, by personal interest, a general surveillance motivation, or desire to gain information for future discussions with others). Email reminders may have appealed most to those individuals who were already interested in their health or health topics in general or who desired to change their diet. Additional research is needed to explicate the motivations for using health websites.

Some topics or message formats in the emails and on the website may have been automatically attention-getting. Prior research showed that leads for online news stories that highlighted conflict and agony produced more selective exposure because danger-conveying signals or empathic sensitivities linked to emotional displays are inherently attention-getting [10]. One such “hardwired” frame in the current study might be personalizing messages by addressing the emails to users by name. Likewise, online messages containing attributes such as animation, ad position, and novelty may elicit an involuntary orienting response [23,24], and the promotion of “new” content in the email reminder may have automatically captured user attention. In future studies, various frames should be tested for improving selective exposure as well as multimedia features such as animation.

### Selective Avoidance of Disease Prevention Websites

Selectivity means that people also can choose to avoid messages that do not interest them [4]. It may be that some participants had low commitment to the trial, low interest in their health and diet, and/or did not find the website attractive and chose to not visit it even when reminded by email. We were not able to detect whether individuals opened the email messages, so we could not directly measure avoidance. Software is now available that can detect when email messages containing hypertext links are opened [25], and it could be used to explore selective avoidance of online content.

### Selective Exposure and Effectiveness of a Disease Prevention Website

In the Cognitive Mediation Model, exposure provokes attention and elaboration or message involvement, which can determine whether the message is effective. Consistent with this perspective, adults who responded to the email messages reported greater pretest-posttest increases in daily fruit and vegetable consumption than adults who did not respond. Internet websites have produced improvements in diet and diet-related behaviors in previous trials [20,26-31], and it appears that strategies that achieve greater exposure to such a website improve their influence [32]. It is notable that the website use and dietary improvements occurred in a multi-ethnic group of adults in rural community environments. Prior studies enrolled mostly white users, and only three were conducted in community environments [30,33,34].

It was surprising that participants who responded to more email reminders did not report larger improvements in fruit and vegetable intake. The motivation to return to the website may have been linked to the same motivations that led to change in dietary behavior (ie, interest in health, plans to change diet), but once there, responders may not have always processed the information effectively. Some processing tendencies for online content (eg, scanning rather than reading carefully) can interfere with learning and may offset gains from increased elaboration of information in the networked structure common on websites [35] (although networked structure may improve understanding [36]). Thus, media exposure is, at best, an imperfect predictor of media effects. Some participants who spent more time on the website may have learned more information and skills and were more persuaded to change their diets, but others may have spent more time because they were not learning enough or were experiencing difficulty altering their diets. Like all media, understanding how the Internet is effective requires understanding both the determinants of exposure and processing of the information once exposure happens.

### The Promise and Pitfalls of Email for Online Disease Prevention Communication

Email reminders may be an especially attractive means of promoting return use of a website. Email is an online function, used by nearly all Internet users [37]. While used equally by all age cohorts, email is the predominant online feature used by older adults [37] relative to other online functions, perhaps explaining why older participants responded most to the email reminders. Email reminders may be especially cost-effective in rural areas, where users are spread over large geographic areas.

However, the multicultural and rural character of the population in the study area made the likelihood of use of an Internet-based intervention somewhat less than might be expected in other areas of the United States. Along with being somewhat behind the general curve of Internet adoption [38], rural residents are frequently underserved in terms of health and medical information and stand to gain from the use of disease prevention websites. They may have more interest in online health information than urban users [38], although in the current sample, Hispanics were less frequent users of the Internet. The challenge remains to meet their needs with effectively designed Internet communication. These findings should generalize to suburban and urban users, too, because email is very popular in all regions of the country [38].

Still, ominous trends in the world of email may limit or diminish its effectiveness. The increasing amount of email advertising, or spam, has created dissatisfaction among Internet users, particularly women, older adults, and more experienced users [39]. Filtering software and other user strategies present barriers to delivering reminders by email. Distraction can interfere with message receipt and effectiveness [4,40], and many Internet users already report that spam makes it difficult to find email messages they desire to read in their inbox, particularly in personal email accounts [39]. Email reminders may be more effective in permission-based marketing circumstances [41,42] where the website provider has an existing relationship with the users (eg, a health insurance provider) and users expect to receive it (but even this may not be acceptable to all users [39]). Internet users also may be experiencing email overload, and the email reminders simply added to the stress of an ever increasing demand to be accessible 24/7 through this medium [43]. Finally, email may be less effective as instant and text messaging and other communication media appear and users become more selective among online media (a phenomenon already witnessed in the young [44]).

### Limitations to the Study

There were limitations in this study. Visits to the website during the days immediately following the email reminders were assumed to be in response to them because we could not detect whether the email messages were opened. The pattern of an abrupt increase in log-ons on the day of transmission certainly is suggestive that most log-ons were in response to these messages. The dietary outcome measure has been validated in previous research, reducing the likelihood of a social desirability bias or demand effect. Still, people who responded to the email messages were self-selected and could have been predisposed to alter their diet or better able to do so. The analysis of change in fruit and vegetable intake is limited by the lower than desired follow-up rate for the survey (62% overall); follow-up was higher among older, married, more educated, white, non-Hispanic participants born outside the region who were living in smaller households with children and who had lived for a shorter time in the region, but it did not differ by treatment group [12].

### Conclusion

It is essential that health providers effectively position their websites to attract use from the intended audience. “Pushing” the content to users registered on a website through routine email messages may be one way of prompting its use. Further research is needed to determine how best to create and present these email messages and to understand the motivations that underlie selective exposure to health websites and the information processing that takes place as users read email messages and navigate websites. Exposure to Internet health communication is a necessary first step to demonstrate its effectiveness in experimental trials and to make providers’ investment in this new communication technology pay off.
